# Effects of the properties of short peptides conjugated with cell-penetrating peptides on their internalization into cells

**DOI:** 10.1038/srep12884

**Published:** 2015-08-10

**Authors:** Ryo Matsumoto, Mina Okochi, Kazunori Shimizu, Kei Kanie, Ryuji Kato, Hiroyuki Honda

**Affiliations:** 1 Department of Biotechnology, Graduate School of Engineering, Nagoya University, Furo-cho, Chikusa-ku, Nagoya 464-8603, Japan; 2 Department of Chemical Engineering, Graduate School of Science and Engineering, Tokyo Institute of Technology, Ookayama, Meguro-ku, Tokyo, 152-8550, Japan; 3 Department of Basic Medicinal Sciences, Graduate School of Pharmaceutical Sciences, Nagoya University, Furo-cho, Chikusa-ku, Nagoya, Aichi 464-8601, Japan

## Abstract

Peptides, especially intracellular functional peptides that can play a particular role inside a cell, have attracted attention as promising materials to control cell fate. However, hydrophilic materials like peptides are difficult for cells to internalize. Therefore, the screening and design of intracellular functional peptides are more difficult than that of extracellular ones. An effective high-throughput screening system for intracellular functional peptides has not been reported. Here, we demonstrate a novel peptide array system for screening intracellular functional peptides, in which both cell-penetrating peptide (CPP) domain and photo-cleavable linkers are used. By using this screening system, we determined how the cellular uptake properties of CPP-conjugated peptides varied depending on the properties of the conjugated peptides. We found that the internalization ability of CPP-conjugated peptides varied greatly depending on the property of the conjugated peptides, and anionic peptides drastically decreased the uptake ability. We summarized our data in a scatter diagram that plots hydrophobicity versus isoelectric point (pI) of conjugated peptides. These results define a peptide library suitable for screening of intracellular functional peptides. Thus, our system, including the diagram, is a promising tool for searching biological active molecules such as peptide-based drugs.

Peptides are important molecules that play a diverse range of roles in the body. Intracellular functional peptides, which play a particular role inside a cell have been used as differentiation-inducing factors^1^ and have applications in theranostics^2^, in particular as peptide drugs. Peptides that exterminate cancer cells by functioning intracellularly include a peptide that induces cell death by restoring the lost function of p16 protein^3^ and a peptide that regulates the cell cycle of cancer cells^4^, among others^5^,^6^,^7^. More recent studies have revealed endogenous, intracellular peptides produced by degrading enzymes (e.g., the ubiquitin-proteasome system)^8^,^9^,^10^, including a peptide that modifies the transcription factor^11^ and another, derived from G_1_/S-specific cyclin D2 that induces cell death^12^. However, since hydrophilic materials such as peptides are typically difficult to internalize into cells, screening of intracellular functional peptides is more difficult than that of peptides that function extracellularly. In other words, intracellular functional peptides are required to possess not only biological activity, such as specific binding to the intracellular biomolecule of interest, but also cell internalization ability. To detect intracellular peptides showing both these activities, an effective screening system for intracellular functional peptides has been aggressively pursued in recent years.

Cell-penetrating peptides (CPPs) have received much attention in recent decades as a tool for delivering various materials with low membrane permeability inside the cell. For example, CPPs can deliver not only biological molecules (e.g., peptides^1^, proteins^13^, nucleic acids^14^) but also comparatively large molecules like liposomes^15^ and nanomagnets^16^. Therefore, CPPs are applicable to a range of research fields, including drug delivery systems (DDS)^17^ and regenerative medicine^18^. Moreover, recently, many researchers have attempted to search intracellular functional peptides by using CPPs; in fact, various peptides have been identified using them^1^,^3^,^4^,^5^,^6^,^7^. The general process for searching for intracellular functional peptides is as follows. First, a target protein is purified and an assay system that allows evaluation of protein function is constructed *in vitro*. Second, peptides that exert their function by binding to the target protein are screened *in vitro* by using various high-throughput methods (e.g., phage display, peptide arrays). Third, the peptides are conjugated to a CPP and are internalized into the cells. Finally, the function of the internalized peptide is evaluated.

However, this conventional method does have some problems: (1) purification of the target protein and construction of the assay system for evaluation of protein function are difficult; (2) peptide activity and/or the internalization ability of CPPs often change when conjugated to each other^19^; (3) even if the function of the peptide is confirmed *in vitro*, it does not guarantee similar functioning in cells because of possible interference factors, including other intracellular molecules. To address these problems, it is important that a CPP-binding peptide library, assuring internalization into cells, is constructed before screening. To the best of our knowledge, such a direct cellular screening system has not been reported.

Peptide array is the one of the most useful tools for analyzing various protein-protein interactions (e.g., antibody^20^, receptor^21^, and cytokine^22^). We have used a cellulose membrane-based peptide array to identify various peptides, including IgG Fc-binding peptide^23^, cell death-inducible peptide^24^, bile acid-binding peptide^25^, and cell-adhesive peptides^26^. Moreover, we have constructed a photo-cleavable peptide array for designing a solubilized peptide library, but not the immobilized library. The library has been used to screen for a peptide that inhibits the function of α-amylase^27^. However, the previously reported peptides influence cell behavior via extracellular interactions. As it is difficult to identify intracellular functional peptides by using existing technologies, we investigated the possibility of constructing a novel screening system for intracellular functional peptides.

In the present study, we describe a novel screening system for intracellular functional peptides, in which the previously reported photo-cleavable peptide array^27^ is combined with CPPs (Fig. 1). Mihara and collaborators have reported the combined use of the photo-cleavable linker and CPPs: Usui *et al.* have demonstrated the array format for monitoring cellular uptake by using a photo-cleavable linker, and the internalization of TMR-(KLAKLAK)_3_ and TMR-Transportan was monitored in real time^28^. Kakiyama *et al.* analyzed the cytotoxicity of TMR-(KLAKLAK)_3_ and TMR-L8K6 by using a photo-cleavable peptide-release system^29^. In these studies, the functions of CPPs (e.g., cellular uptake, cytotoxicity) were investigated using a photo-cleavable linker^29^. In the present study, we developed a peptide array-based screening system for intracellular functional peptides. We also conjugated peptides of various lengths with CPPs to identify properties of conjugated peptides that enable them to be internalized into cells. We synthesized 31 kinds of CPP-binding peptide arrays on cellulose membranes and investigated the change in cellular uptake depending on the properties of these peptides. This is the first report of an effective screen for intracellular functional peptides by identifying a peptide library that can be internalized into cells with CPP tags.

## Results and Discussion

### Construction of the intracellular functional peptide screening system

**Figure 1:**
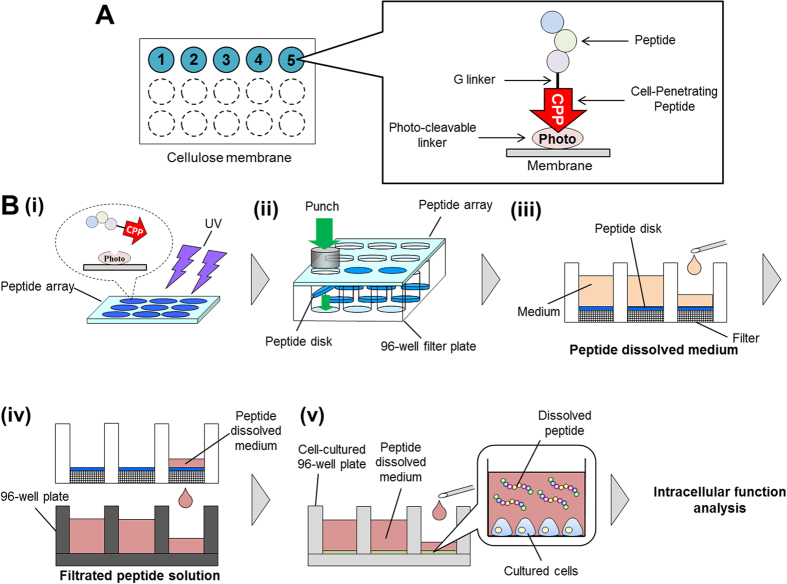
Schematic representation of peptide array-based intracellular functional peptide screening. (**A**) Starting from peptide synthesis on cellulose membrane by peptide array synthesizer, this figure is an overview of how a peptide is spotted on the cellulose membrane. The word “Photo” means photo-cleavable linker. (**B**)(i) Peptides spotted on a membrane were cut by UV irradiation. (ii) Each peptide spot was punched out as a disk and placed in one well of a 96-well plate. (iii) These peptides were dissolved by adding the medium, and (iv) this solution was filtered. (v) Filtered peptide-containing solutions were added to a second 96-well plate containing cultured cells. Intracellular functions impacted by the peptides were analyzed.

For constructing the intracellular functional peptide screening system, photolinker-peptide arrays and cell-penetrating peptides (CPPs) were combined. We chose octaarginine (R8) as a CPP of this screening system and synthesized it on photo-cleavable peptide arrays. Peptides were conjugated to R8 via a glycine (G) linker (Fig. 1A). To cleave these CPP-peptide libraries from the arrays, they were irradiated by UV (365 nm) for few hours (Fig. 1B(i)). After UV irradiation, each spot on the array was punched out (about 6 mm in diameter) and placed in a single well of a 96-well plate along with a filtration filter (Fig. 1B(ii)). Fresh culture medium was added to the well; once the peptide dissolved, the medium was filtered using vacuum filtration to get rid of insoluble matter (Fig. 1B(iii,iv)). The medium was transferred to the wells of another 96-well plate containing cultured HeLa cells, and various intracellular functional analyses were performed (Fig. 1B(v)).

### Quantification of cleaved peptide by changing UV irradiation time

**Figure 2:**
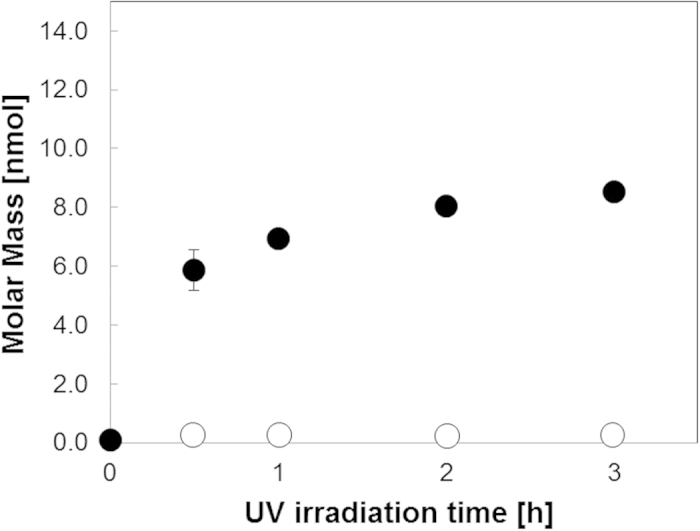
Amount of peptide cleaved versus UV (365 nm) irradiation time. Peptides on the membrane spots (n = 3) were cleaved by UV irradiation (365 nm). Black circles denote Fluorescein-GABA-RRRRRRRR-Photo-cleavable linker, which can be cleaved by UV. White circles denote Fluorescein-GABA-RRRRRRRR-11-aminoundecanoic acid, which cannot be cleaved by UV (negative control). Peptide concentration was determined by measuring the amount of fluorescence. The standard curve of the purified Fluorescein-GABA-RRRRRRRR was used to determine the concentration of the cleaved peptide.

We first determined the optimum UV irradiation time for peptide cleavage (Fig. 1B(i)). Fluorescein-GABA (γ-aminobutyric acid)-RRRRRRRR (R8) peptide was synthesized on the spots with and without the photo-cleavable linker. After R8 peptide was synthesized, NHS-fluorescein was conjugated on the N-terminal of peptide via GABA. As shown in Fig. 2, Fluorescein-GABA-R8 on the photo-cleavable linker was cleaved from the arrays within 3 h of irradiation and the maximum amount of cleaved peptide was 8.52 nmol/spot, which corresponds to 30.4% of the total peptides synthesized in a single spot of the cellulose membrane^27^,^30^. We previously reported^27^ that UV irradiation released 15 nmol/spot octamer peptide, and up to 54% peptide cleavage can be achieved. The reason for low cleavage efficiency by UV irradiation in the present study is due to the synthesis of longer peptide. It was likely that longer time or more steps for peptide synthesis allows the cleavage of photolinker, resulting in undesirable release of peptides during synthesis.

Fluorescein-GABA-R8 on 11-aminoundecanoic acid was not cleaved from the arrays within 3 h of irradiation because this linker did not have a photo-cleavable site (Fig. 2). We measured the molar mass of cleaved peptides by using MALDI-TOF-MS and confirmed that the cleaved peptide was Fluorescein-GABA-R8 (Fig. S1). Because previous studies have evaluated the function of intracellular peptides in living cells within the range of 10–50 μM^3^,^4^,^6^,^7^, we decided to use 250 μL cell culture medium to dissolve the cleaved peptides. The resulted concentration of peptide was expected to be sufficient to screen intracellular functional peptides.

### Internalization of the cleaved R8 from the arrays into cells

**Figure 3:**
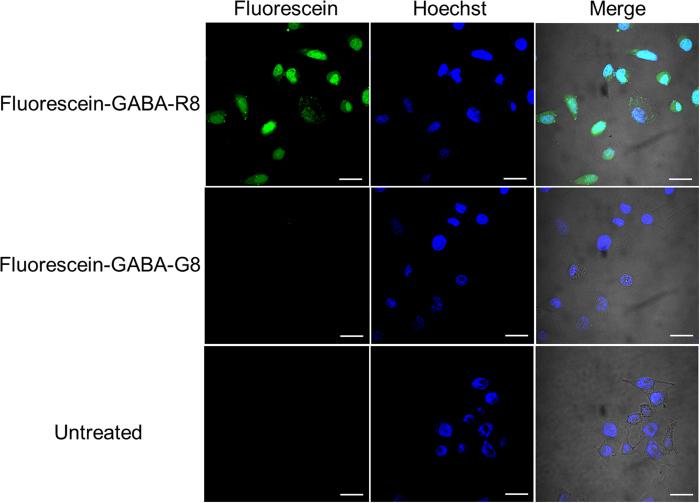
Internalization of the detached peptide by HeLa cells. Confocal microscopy analysis of HeLa cells was conducted after incubating the cells at 37 °C for 3 h with Fluorescein-GABA-R8, Fluorescein-GABA-G8 as a negative control, and untreated control. About 1.5 × 10^5^ HeLa cells were seeded in 35-mm glass-bottom dish 24 h before the experiment. Scale bar = 30 μm.

To test if the members of the CPP binding-peptide library cleaved from the arrays can be internalized into living cells, we evaluated the internalization of Fluorescein-GABA-R8. The peptide-synthesized spots on the membrane were punched out as disks (diameter, 6 mm), the disks were dropped into a low-binding centrifuge tube, and DMEM was added to the tube. The internalization of the peptide was examined by confocal microscopy after 3 h of incubation of the peptide with HeLa cells at 37 °C. As shown in Fig. 3, Fluorescein-GABA-R8 was internalized into the cells and localized to both cytoplasm and nucleus, as reported elsewhere^31^. In contrast, no green fluorescence was observed when the negative control peptide, Fluorescein-GABA-GGGGGGGG (G8), was examined. The negative control peptide was not internalized into cells because G8 does not have a cell-penetrating property.

Our approach was advantageous as compared to conventional peptide synthesis using resin with regard to adaptability to the robotized high-throughput screening system, fewer steps required for peptide preparation, and reduction in cost per peptide to <1% of that using resin^32^. Additional steps are required to recover peptides from the resin, such as cleavage of peptide from resin by strong acid, refinement of peptide in strong acid by ice ether (e.g., diethyl ether), and lyophilization of peptide in ether. On the other hand, UV irradiation is sufficient to recover peptides from the membrane in our method. Furthermore, the number of screening libraries, which are more important for first screening than the amount and/or the purity of the peptides, can be easily increased. For these reasons, we believe that our strategy is better suited than resin synthesis, particularly for first screening.

### Modulation of cellular uptake properties by adding tripeptides to R8

**Figure 4:**
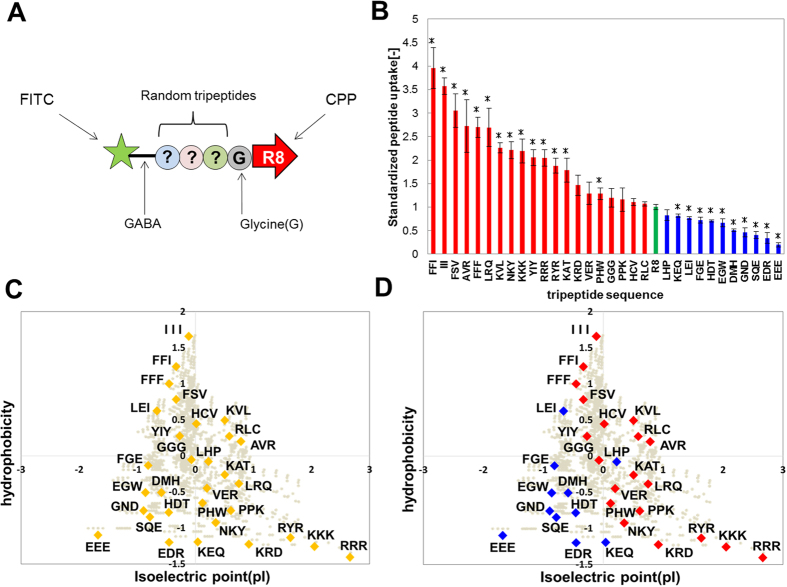
Modulation of released peptide uptake ability by adding tripeptide sequences to R8. The uptake of released tripeptide conjugated R8 was measured with a multi-plate reader. (**A**) This figure is a schematic of the peptide motifs used in intracellular uptake measurement. (**B**) Uptake of tripeptide-conjugated R8 compared to that of R8 alone (green bar). Red bars represent tripeptides that showed enhanced cellular uptake ability, compared to the control. Blue bars represent tripeptides that showed decreased cellular uptake, compared to the control. On the y-axis is the standardized value of the result measured with the multi-plate reader. All measurement scores were normalized to the control (R8) as 1.0. (**C**) This figure is a chart of all the tripeptide sequences classified by hydrophobicity versus isoelectric point (pI). The values of these indices were normalized. Gray circles denote all tripeptides. (**D**) Color classification based on the results of (**B**). Means ± SD of three experiments are shown. *p < 0.05.

Once the screening system for intracellular functional peptides using a CPP tag was constructed (Figs 2 and 3), we screened peptides which could be internalized into cells, as a biological application of the developed screening system. Thus, we identified the properties of conjugated peptides that enabled them to be internalized into cells, by conjugating peptides of various lengths with CPPs. We examined the internalization of CPP-conjugated peptide with a tripeptide inserted into Fluorescein-GABA-R8. Thirty-one different kinds of tripeptide were inserted between GABA and R8 in Fluorescein-GABA-R8, as shown in Fig. 4A. A glycine residue was used as the linker between the tripeptides and R8. To assay internalization properties, we determined the degree of internalization in HeLa cells with a microplate fluorometer after 6 h of incubation of each CPP-conjugated tripeptide with the cells at 37 °C. The results revealed that the degree of internalization varied depending on the tripeptide sequence (Fig. 4B). For example, the uptake of FFI-conjugated R8 was 4-fold higher than that of R8 only (control), whereas that of the EEE-conjugated R8 was very low.

In summary, we quantified the chemical properties of the inserted tripeptides by using two indices – hydrophobicity and isoelectric point (pI). The data for all tripeptides analyzed were plotted in a scatter diagram (Fig. 4C). As shown in Fig. 4D, the tripeptides with high uptake compared to the control had high hydrophobicity or cationic charge combined with low hydrophobicity (hydrophilicity) (Fig. 4D). In addition, we found that the uptake of R8 was decreased by adding anionic tripeptide sequences (Fig. 4D). Tripeptides with low hydrophobicity and low pI had the lowest uptake. No correlation between the amount of cellular uptake of the peptides and their concentration was apparent (R^2^ = 0.045, Fig. S2).

### Modulation of cellular uptake properties by adding pentapeptides to R8

**Figure 5:**
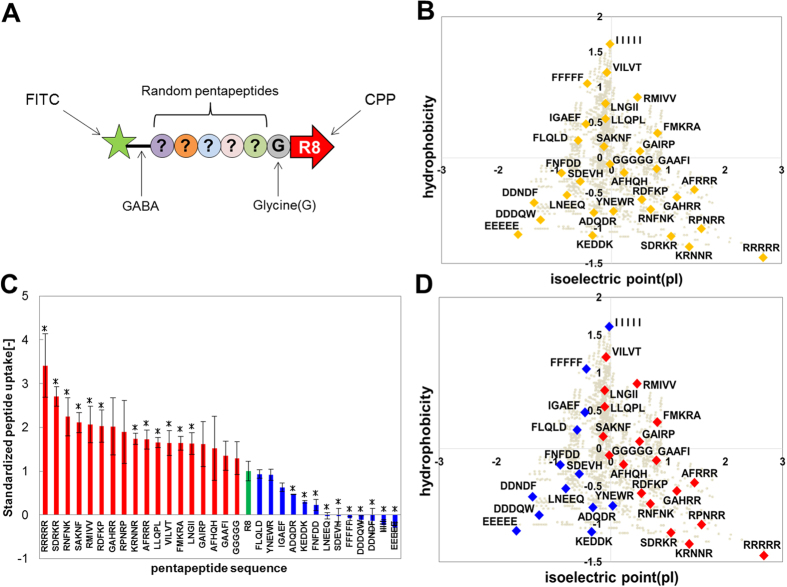
Modulation of detached peptide uptake ability by adding pentapeptide sequences to R8. The uptake of the released pentapeptide-conjugated R8 was measured with a multi-plate reader. (**A**) This figure shows the peptide motifs assessed for intracellular uptake. (**B**) This figure shows the 31 pentapeptides classified by hydrophobicity versus isoelectric point (pI). The values of these indices were normalized. Gray circles denote all pentapeptides. (**C**) The result of uptake of pentapeptide-conjugated R8 compared to that of R8 only (green bar). Red bars denote pentapeptide sequences that enhanced cellular uptake, compared to that of the control. Blue bars represent sequences that decreased cellular uptake ability, compared to that of control. On the y-axis is the standardized value of the result measured with the multi-plate reader. All measurement scores were normalized to the control (R8) as 1.0. (**D**) Color classification based on the results of (**C**). Means ± SD of three experiments are shown. *p < 0.05.

We next investigated whether the properties of tripeptides (Fig. 4) can be generalized to longer peptides. Thirty-one pentapeptides were selected and plotted on a scatter diagram to describe their hydrophobicity and pI (Fig. 5A,B). Cellular uptake properties varied greatly upon addition of pentapeptides to R8 (Fig. 5C). For instance, the uptake of RRRRR-conjugated R8 was 3.5-fold higher than that of R8 alone, whereas that of EEEEE-conjugated R8 was very low. Similar to the results obtained for tripeptides (Fig. 4D), the cellular uptake properties of R8 were enhanced in pentapeptides with high hydrophobicity, or cationic charge and low hydrophobicity. The less-hydrophobic-and-low-pI pentapeptides showed low uptake (Fig. 5D). No correlation was observed between cellular uptake and peptide concentration (R^2^ = 0.1294, Fig. S3), which agrees with our findings for tripeptides (Fig. S2). In addition, some previous findings were also explained by our results (Figs 4 and 5); the internalizing efficiency of R8 was considerably increased by addition of FFFF, a hydrophobic-rich peptide segment^33^ as well as R16 and R12, which are richer in cationic-charge density than R8, thus achieving higher internalizing efficiency than R8 alone^34^. Based on these results, we concluded that the tendency for the R8 peptide to be internalized into a cell can be described using hydrophobicity and pI as indices.

As shown in Fig. 5D, some peptides, including FFFFF and IIIII, which have high hydrophobicity, possessed significantly low cellular uptake properties (Fig. 5D). It was previously reported that R8-conjugated FFFF enhanced uptake ability, but R8-conjugated FFFFFF did not dissolve into PBS^33^. Therefore, we examined the solubility of the peptides, R8-conjugated FFFFF and IIIII. Not surprisingly, these peptides failed to dissolve in our medium (Fig. S3). Therefore, it is reasonable to conclude that the low cellular uptake of these peptides is due to low solubility.

The less-hydrophobic-and-low-pI peptides showed lowest cellular uptake (Figs 4D and 5D). Thus, less-hydrophobic-and-low-pI peptides were not suitable for internalization by conjugation with R8. Eliminating unsuitable peptide sequences from the library of the conjugated peptides prior to the experiments will improve the effectiveness of screening (Figs 4D and 5D). Therefore, we suggest that the peptide library classified by the two indices is suitable for screening and designing intracellular functional peptides.

Studies to date have focused mainly on CPPs, with a few investigating the mechanism of their internalization into cells^35^. It has been shown that different CPPs involve various mechanisms such as endocytosis^36^, micropinocytosis^37^, and membrane disruption^38^, while other studies included modified CPPs to improve their internalization ability^33^,^39^, such as adding highly hydrophobic sequences to CPPs^33^. In the present study, however, we focused on conjugating CPPs with novel candidates for intracellular functional peptides. We synthesized these candidate peptides using the peptide array system, and systematically investigated the relation between their properties (hydrophobicity and pI) and the resultant internalization ability (Figs 4 and 5). To our knowledge, no previous study investigated the uptake tendency of functional peptides. Therefore, our results would provide new information that would be useful, with respect to not only CPPs, but also novel intracellular functional peptides.

In conclusion, we synthesized peptides that combined photo-cleavable peptide arrays and cell-penetrating peptides (CPPs) and investigated how CPP uptake varied depending on the property of the conjugated peptides. We also proposed a scatter diagram for predicting peptide properties. Our proposed system, including the diagram, holds a lot of promise as a tool to address the need for a peptide library to screen intracellular functional peptides.

## Materials and Methods

### Synthesis of photo-cleavable peptide arrays

The synthesis of photo-cleavable peptide arrays was reported previously^27^. A cellulose membrane (grade 542; Whatman, Maidstore, UK) was activated using β-alanine as the N-terminal basal spacer. A Fmoc-Photo-Linker (sc-294977A, SANTA CRUZ, USA) was used as photo-cleavable linker for Fmoc peptide synthesis. The linker was conjugated between candidate peptides and cellulose. Activated Fmoc amino acid (0.5 M) was spotted on the membrane by using a peptide auto-spotter (ASP222; Intavis, Cologne, Germany), following the manufacturer’s instruction with some modifications. After adding the first residue, the remaining amino groups were blocked twice with 5% acetic anhydride for 15 min. At each elongation step, the membrane was deprotected using 20% piperidine and then washed thoroughly with N,N′-dimethylformamide, followed by a wash with methanol. For synthesis of fluorescein on N-terminal each peptides, NHS-fluorescein (46409; Pierce Biotechnology, USA) was used with N,N′-diisopropylethylamine (550043; Sigma-Aldrich, USA) in N-methylpyrrolidone (NMP). After final deprotection, the side-chain protecting groups were removed for 2.5 h by using a mixture of trifluoroacetic acid (TFA, A00025; Watanabe, Japan), *m*-cresol (034-04646; Wako, Japan), 1,2-ethanedithiol (A00057; Watanabe), thioanisole (T0191; Tokyo Chemical Industry, Japan) at a ratio of 40:1:3:6. Finally, the membrane was washed thoroughly with diethylether, methanol and ultrapure water, consecutively. Three spots for each peptide sequence were deposited on each membrane. We confirmed the quality of peptides synthesized using the peptide auto-spotter, by analyzing each residue of peptides with bromophenol blue (BPB), and performed a spot check by MALDI-TOF-MS.

### Release of peptides from the photo-cleavable peptide arrays

Each peptide on the photo-cleavable peptide arrays was cleaved from the solid phase by irradiation with UV at 365 nm. The peptide arrays were dried completely at room temperature, and irradiated with UV at 365 nm for 2 h by using a transilluminator (DT-20LCP; Atto, Japan). After irradiation, each spot on the array was punched using a biopsy punch (diameter, 6 mm; KAI Corp., Japan). Each of the resulting peptide-containing disks (peptide spots) was placed in a single well of a 96-well plate with a filtration filter (MSRLN0410; Merck Millipore, Germany) and 250 μL of Dulbecco’s modified Eagle medium (DMEM) supplemented with 2% fetal bovine serum (FBS; Life Technologies, Carlsbad, CA, USA) and 1% Penicillin/Streptomycin (Life Technologies). To dissolve the peptide, the medium was vigorously agitated for 15 min with a sonicator. After dissolving each peptide, the medium containing dissolved peptide was filtered into a 96-well plate by vacuum filtration (MultiScreen HTS Vacuum Manifold; Merck Millipore) to remove various insoluble material, and was subsequently used for the intracellular uptake assay.

### Quantification of cleaved peptide from photo-cleavable peptide arrays

Fluorescein-GABA (γ-aminobutyric acid)-RRRRRRRR (R8) peptide spots after UV irradiation were placed in a single well of a 96-well plate with a filtration filter; 100 μL of PBS was added. After dissolving this peptide, PBS containing the dissolved peptide was filtered by vacuum filtration to remove various insoluble matters, and then transferred to another 96-well plate. Fluorescence intensity was subsequently measured by microplate fluorometer (Fluoroskan Ascent; Thermo Scientific, USA). The concentration of this peptide was determined on the basis of the standard curve of the synthesized fluorescein-GABA-R8 (purity >95%). This peptide was purchased from Sigma-Aldrich (USA).

### Cell culture

HeLa cervical cancer cells (JCRB9004; JCRB Cell Bank, Osaka, Japan) were maintained on 10-cm dishes cultured in DMEM supplemented with 10% FBS, and 1% Penicillin/Streptomycin. Cells were cultured in a humidified 5% CO_2_ incubator at 37 °C to approximately 80% confluence.

### Confocal microscopy

HeLa cells (1.5 × 10^5^ cells/well) were plated in 35-mm glass-bottomed dishes (3970-035; Iwaki, Japan) and cultured for 24 h. The cells were washed with DMEM supplemented with 2% FBS, and 1% Penicillin/ Streptomycin before incubation with the peptides in medium. After incubation for 3 h, the incubated medium was removed and the cells were washed three times with PBS. The cells were stained with 10 μg/mL Hoechst 33258 (Dojindo, Japan) for 15 min and subsequently were washed three times with PBS and twice with HBSS buffer for the next observation. The intracellular uptake of peptides was immediately observed, without fixation of the cells, using a confocal laser-scanning microscope (FV1000-D; Olympus, Japan).

### Intracellular uptake assay

Hela cells (2.5 × 10^4^ cells/well) were plated in wells of black 96-well glass-bottomed plates (655892; grainer bio-one, Germany) and cultured for 24 h. The cells were washed twice with DMEM supplemented with 2% FBS, and 1% Penicillin/Streptomycin before incubation with the peptides. After incubation for 6 h, the peptide-containing medium was removed, and the cells were washed gently five times with PBS. The fluorescence intensities of the wells were immediately measured using a microplate fluorometer. The uptake amount of each peptide was determined on the basis of the standard curve of the synthesized Fluorescein-GABA-R8 (purity >95%).

### Creation of peptide scatter diagram

For generating the comprehensive tripeptide scatter diagram, we chose two indices; hydrophobicity^40^ and isoelectric point (pI)^41^, which are basic indices of the various properties of amino acids. The values of these indices were initially normalized. All tripeptides in the scatter diagram were plotted on the basis of the following equation:


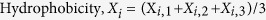



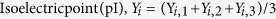


Where X_*i,*1_, X_*i,*2_, and X_*i,*3_ are the hydrophobicity value of the 1st, 2nd, and 3rd amino acid from the N-terminal end of the peptide, *i*, and Y_*i,*1_, Y_*i,*2_, and Y_*i,*3_ is the isoelectric point (pI) value of the same amino acid of peptide, *i*, respectively. Thus, X_*i*_ and Y_*i*_ indicate the average hydrophobicity and pI of constituent amino acids, respectively, of the peptide *i*. For the pentapeptide library, only 31 pentapeptides were also plotted on this scatter diagram by using a similar technique of average hydrophobicity and pI.

### Statistical analysis

Data are presented as mean values and standard deviation (SD), and a Student’s t-test was used for evaluating statistical significance for comparison. A value less than 0.05 (p < 0.05) indicated statistical significance.

## Additional Information

**How to cite this article**: Matsumoto, R. *et al.* Effects of the properties of short peptides conjugated with cell-penetrating peptides on their internalization into cells. *Sci. Rep.* **5**, 12884; doi: 10.1038/srep12884 (2015).

## Supplementary Material

Supporting Information
