# Supplementary material for: Effects of the properties of short peptides conjugated with cell-penetrating peptides on their internalization into cells
Source: Sci Rep. 2015 Aug 10;5:12884. doi: 10.1038/srep12884 (PMC4530456; doi:10.1038/srep12884)
Supplement: Supporting Information [file srep12884-s1.pdf]

Supporting Information

Effects of the properties of short peptides conjugated with cell-penetrating peptides on their internalization into cells

Ryo Matsumoto[1], Mina Okochi[2], Kazunori Shimizu[1], Kei Kanie[1,3], Ryuji Kato[1,3], Hiroyuki Honda[1,*]

[1]Department of Biotechnology, Graduate School of Engineering, Nagoya University, Furo-cho, Chikusa-ku, Nagoya 464-8603, Japan
[2]Department of Chemical Engineering, Graduate School of Science and Engineering, Tokyo Institute of Technology, Ookayama, Meguro-ku, Tokyo, 152-8550, Japan
[3]Department of Basic Medicinal Sciences, Graduate School of Pharmaceutical Sciences, Nagoya University, Furo-cho, Chikusa-ku, Nagoya, Aichi 464-8601, Japan

Table of Contents

Figure S1 Detection of Fluorescein-GABA-RRRRRRRR by MALDI-TOF-MS

Figure S2 Relationship between uptake and tripeptide-conjugated R8 concentration

Figure S3 Relationship between uptake and pentapeptide-conjugated R8 concentration

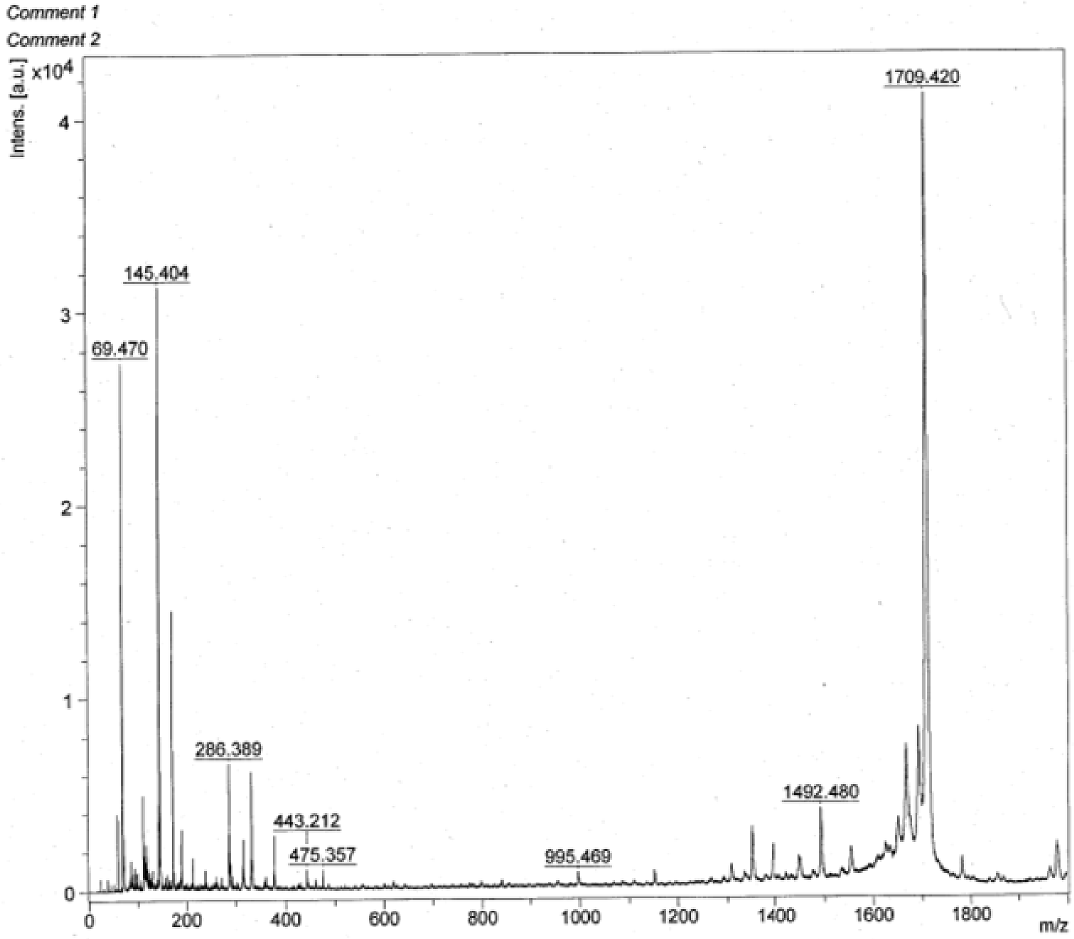

Fig. S1. Detection of Fluorescein-GABA-RRRRRRRR by MALDI-TOF-MS
Fluorescein-GABA-RRRRRRRR MW; 1709.98. This figure indicates that the synthesis of Fluorescein-GABA-RRRRRRRR progressed correctly.

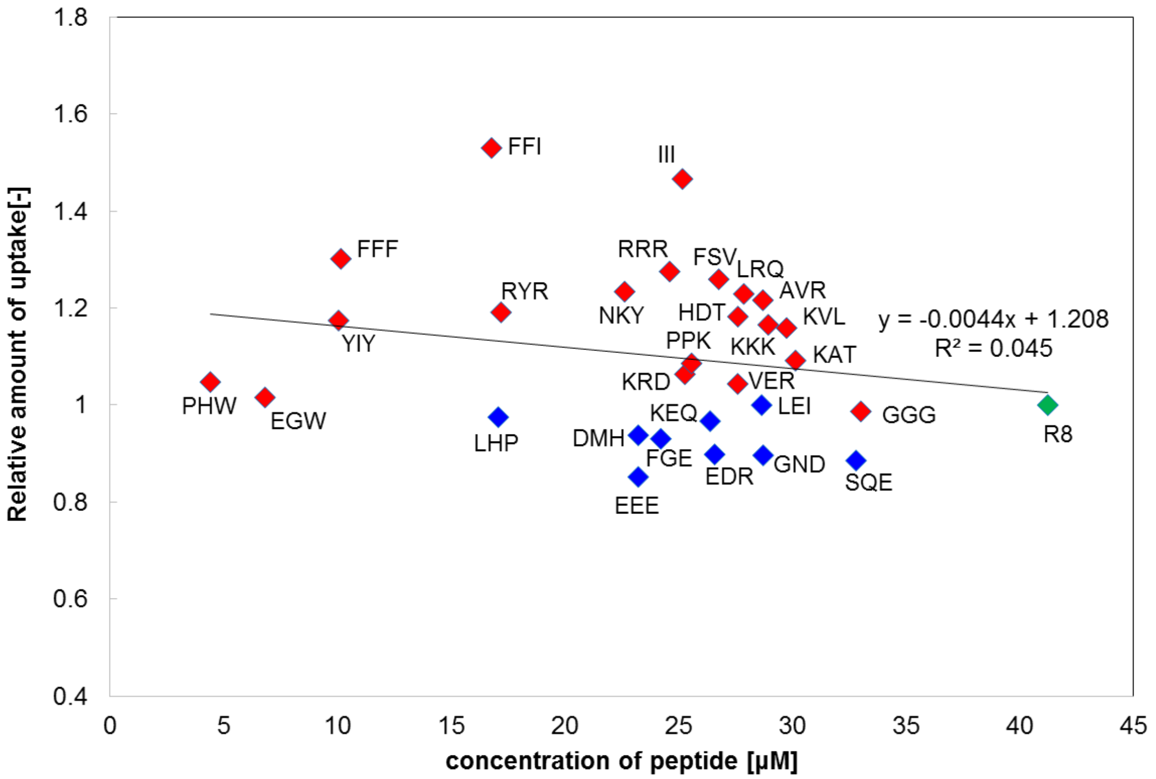

Fig. S2. Relationship between uptake and tripeptide-conjugated R8 concentration

Peptide concentration was determined using a microplate fluorometer, based on the standard curve of Fluorescein-GABA-RRRRRRRR. The relative amount of uptake was determined by the following equation: (the score of uptake amount of each tripeptide-conjugated R8) / (the score of uptake amount of R8). The color scheme is based on Fig. 4. RLC- or HCV-conjugated R8 was eliminated from this figure because they did not dissolve in the medium because of the self-quenching of fluorescence.

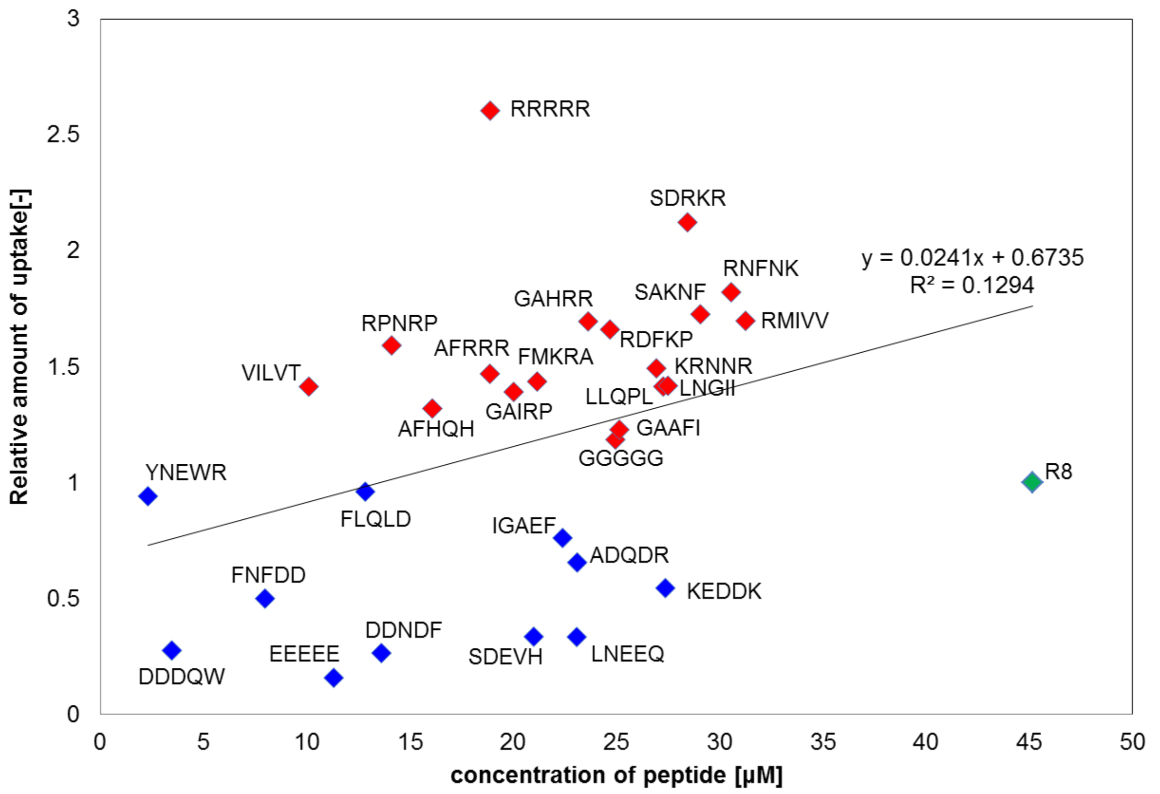

Fig. S3. Relationship between uptake and pentapeptide-conjugated R8 concentration
Peptide concentration was determined using a microplate fluorometer, based on the standard curve of Fluorescein-GABA-RRRRRRRR. The relative amount of uptake was determined by the following equation: (the score of uptake amount of each pentapeptide-conjugated R8) / (the score of uptake amount of R8). The color scheme is based on Fig. 5. FFFFF- or IIIIII-conjugated R8 was eliminated from this figure because they failed to dissolve in the medium.
